# Polyphenolic and Anthocyanin Responses to Postharvest Fungal Pathogen Infection in Purple and Green near Isogenic Pepper (*Capsicum annuum*) Lines

**DOI:** 10.3390/antiox14121440

**Published:** 2025-11-29

**Authors:** Zsófia Kovács, Gábor Csilléry, Hussein Gehad Daood, Katalin Posta, Janka Bedő, Kitti Andrea Tóth-Lencsés, Anikó Veres, Antal Szőke, Ákos Tarnawa, Ákos Juhász

**Affiliations:** 1 Institute of Genetics and Biotechnology, Department of Genetics and Genomics, Hungarian University of Agriculture and Life Sciences (MATE), 2100 Gödöllő, Hungary; kovacs.zsofia@uni-mate.hu (Z.K.); bedo.janka@uni-mate.hu (J.B.); toth-lencses.andrea.kitti@uni-mate.hu (K.A.T.-L.); veres.aniko@uni-mate.hu (A.V.); szoke.antal@uni-mate.hu (A.S.); 2 PepGen Kft., 1114 Budapest, Hungary; csillerygabor48@gmail.com; 3 Food Analysis Laboratory, Hungarian University of Agriculture and Life Sciences (MATE), 2100 Gödöllő, Hungary; daood.hussein@uni-mate.hu; 4 Agribiotechnology and Precision Breeding for Food Security National Laboratory, Department of Microbiology and Applied Biotechnology, Institute of Genetics and Biotechnology, Hungarian University of Agriculture and Life Sciences (MATE), 2100 Gödöllő, Hungary; posta.katalin@uni-mate.hu; 5 Institute of Agronomy, Department of Agronomy, Hungarian University of Agriculture and Life Sciences (MATE), 2100 Gödöllő, Hungary; 6 Institute of Genetics and Biotechnology, Department of Microbiology and Applied Biotechnology, Hungarian University of Agriculture and Life Sciences (MATE), 2100 Gödöllő, Hungary; juhasz.akos@uni-mate.hu

**Keywords:** anthocyanins, *Capsicum annuum*, storage pathogens, *Botrytis*, polyphenols, HPLC

## Abstract

Postharvest fungal pathogens, such as *Botrytis cinerea*, *Alternaria alternata* and *Fusarium culmorum*, pose major challenges for pepper (*Capsicum annuum*) storage and shelf-life. To explore the basis of induced resistance, near isogenic lines (NILs) differing in pigmentation (green vs. purple fruits and their red ripe counterparts) were artificially inoculated and evaluated for disease severity by phenotyping and by qPCR, and metabolite composition by spectroscopy and by HPLC. Infection severity was strongly dependent on whether purple or green NILs were infected and on ripening stage: economically ripe fruits were most susceptible to *B. cinerea*, whereas biologically ripe fruits displayed higher infection rates with *A. alternata*. In the case of *B. cinerea* infection, detailed HPLC analysis revealed that chlorogenic acid and p-coumaric acid were identified as infection-responsive metabolites after analyzing the metabolite changes upon infection. Total anthocyanin content and delphinidin derivatives measured decreased upon infection; however, this effect was not significant in correlation with the infection severity, indicating that *B. cinerea* infection in low, moderate or severe amounts will lead to the degradation of these compounds. Overall, our findings indicate that anthocyanin accumulation alone did not confer resistance to *B. cinerea* in pepper, whereas specific hydroxycinnamic acids emerged as infection-responsive markers.

## 1. Introduction

Solanaceous crops play a vital role not only in terms of human nutrition but from an economical point of view as well. According to FAOSTAT, tomatoes ranked first, with 186 million tons, whereas peppers ranked seventh, with almost 37 million tons, amongst the vegetables produced worldwide. From a dietary point of view, peppers are an excellent source of vitamin C, flavonols, flavones and carotenoids, which not only benefit human health but may also help reduce postharvest losses.

According to estimates, postharvest losses of the global fruit and vegetable production range from 28 to 55% [1], of which 10 to 30% can be attributed to losses caused by different microorganisms [2]. Peppers are generally characterized by a short shelf-life; to overcome this issue, different approaches have been developed, i.e., exogenous application of melatonin [3], brassinosteroids [4], glutathione [5], usage of edible coatings [6,7,8] or the application of different treatments such as steam or microwave [9], but the most prevalent preservation technique is still low-temperature storage [10,11,12]. However, pepper fruits are susceptible to chilling injuries below temperatures of 7–9 °C [13]; further, cold temperatures do not necessarily inhibit the growth of fungal pathogens. *Botrytis cinerea* can grow within a temperature range of 0.5–32 °C [14] and *Alternaria alternata* within a range of 0 to 35 °C [15]. *B. cinerea* is amongst the most researched fungal pathogens causing pre- and postharvest rotting, leading to the development of gray mold disease [1,16,17]. Solanaceus crops are also greatly affected by *A. alternata,* which typically causes black mold, and *Fusarium* spp., causing internal fruit rot in peppers [18,19,20,21].

Upon infection, these pathogens secrete cell-wall-degrading enzymes such as pectinases, endopolygalacturonases, cellulases, cutinases and proteases that facilitate host colonization [22,23]; however, susceptibility to infection depends on an array of factors; for example, the ripening stage affects pathogen growth [24], and during maturation, metabolic processes undergo drastic changes: the fruit starts to soften, ethylene and sugars accumulate, the amount of polyphenolics declines and the pH shifts, all of which favors pathogen colonization [25,26,27,28]. Nevertheless, the accumulation of sugars and antifungal proteins during the ripening of grapes has been correlated with resistance to *B. cinerea* [29].

Phenolic compounds—particularly flavonoids and anthocyanins—have attracted considerable interest as they are central to plant defense. Anthocyanins, besides their well-known antioxidant properties, have been implicated in direct antifungal activity by altering fungal cell walls and membrane permeability [30] and consequently inhibiting spore germination and germ tube growth [31]. In tomato, elevated anthocyanin levels have been associated with enhanced resistance to *B. cinerea* infection [32,33], supporting the hypothesis that these pigments may play a similar protective role in peppers. This idea was already validated with *Phytophthora capsici,* through a study in which purple pepper leaves were shown to be slower to develop symptoms [34]. With the emergence of pathogen strains resistant to different fungicides [35,36], there is a need for safer, natural antifungal compounds such as anthocyanins that could provide an eco-friendly alternative for postharvest disease management.

Pepper cultivars display considerable variation in anthocyanin content, especially at the stage of economical ripeness [37,38]. As the fruit matures to the biologically ripe stage, anthocyanin levels typically decline, while carotenoid concentrations increase [39]. This shift in pigment composition is accompanied by changes in the broader phenolic profile, which may influence the fruit’s susceptibility to postharvest pathogens [40,41]. Nevertheless, the role of anthocyanins in conferring resistance against *A. alternata* and *Fusarium* spp. in pepper remains poorly documented.

Understanding how pigment composition, phenological stage and genotype interact to influence fungal resistance could provide valuable insights for breeding programs and for the development of targeted postharvest management strategies. To address this gap, the present study investigates the response of two near isogenic pepper lines—one purple and one green at their economical ripeness—to *B. cinerea*, *A. alternata* and *Fusarium culmorum* infection at both economically and biologically ripe stages. Disease progression was assessed by phenotyping and by qPCR, while changes in metabolite composition were analyzed by spectrophotometer and by HPLC.

We hypothesized that fruits with higher anthocyanin and therefore higher total polyphenol contents would show greater tolerance to these fungal infections.

## 2. Materials and Methods

### 2.1. Plant Materials

Samples of *Capsicum annuum* var. *cerasiforme* NILs were collected from greenhouses in Szentes, Hungary, maintained by Gábor Csilléry. One of the NILs is purple (labeled as Purple or P) at economical ripeness and matures into a red color (labeled as Red from Purple or RP); the other NIL is green (labeled as Green or G) at economical ripeness and matures into a red-colored fruit (labeled as Red from Green or RG). Fruits both at economical ripeness (ER) and at full biological ripeness (BR) were selected based on consistent maturity levels, uniform appearance and no visible physical defects. Upon harvest, the fruits were surface sterilized with 3% *v*/*v* sodium hypochlorite for 20 min and rinsed with sterile water; then, they were used immediately for the pathogen growth and inoculation tests.

### 2.2. Pathogen Assays

#### 2.2.1. Pathogen Growth Tests In Vitro

For growth tests, pathogens—*B. cinerea* NCAIM (National Collection of Agricultural and Industrial Microorganisms) F.0075 and our own isolates of *F. culmorum* and *A. alternata* isolated and identified from decayed tomatoes and maintained in our laboratory—were grown on potato dextrose agar (PDA) plates (VWR^®^ Chemicals, Leuven, Belgium) supplemented with 50% pepper juice, made by homogenizing approximately a kilogram of deveined and deseeded pepper pods followed by centrifugation and filtration. For the control, plain PDA was used. Five µL spore suspension was pipetted into the center of the plates. The spores were collected from 7 to 10 day-old colonies and set to a spore count of 2 × 10^6^/mL with physiological salt solution with a hemocytometer. The plates were kept at 28 °C and mycelia growth was measured in every 8–10 h for a period of approximately 3 days. Each plate was made in three replicates.

#### 2.2.2. Artificial Infection Studies In Vivo with *B. cinerea*, *F. culmorum* and *A. alternata*

Parallel to the in vitro growth tests, artificial infection studies were carried out. For this, the pepper fruits were divided into four color groups corresponding to the two phenophases. Each batch contained 10 fruits, and each fruit was wounded with a sterile scalpel in a single location, forming an ‘X’ of 2 mm wide and 2 mm deep, onto which 5 µL spore suspension as described above was pipetted. Non-inoculated but wounded fruits (mock-infected) served as the control. Fruits were kept at 25 °C in a Memmert type HPP 260 growth chamber in sterile containers and aerated in every 24 h. The samples were evaluated at 13 days post infection (dpi). The fruits evaluated were those that exhibited browning, sunken lesion or mycelia growth at the point of inoculation. For the phenotypic evaluation of infection severity (%), to calculate the size of the lesions or mycelia growth compared to the size of the fruits’ surface area, we used Fiji (ver. 2.17.0), an image processing software [42].

To complement the phenotypic evaluation, further quantification of the pathogen growth was measured using qPCR approach. For qPCR, the inoculated fruits were freeze-dried and homogenized. DNA was extracted using a Macherey-Nagel Nucleospin Plant II Kit, Düren, Germany. For the qPCR 50 ng template, DNA was used to determine the ratio of fungal pathogens to the pepper genomic DNA. Primer pairs were designed based on the following sequences, for *A. alternata* GenBank accession LR778188.1 (*A.alt* F: CGAATGTTTGAACGCACATTG, *A.alt* R: CGCTCCGAAACCAGTAGG), for *F. culmorum* GenBank accession LT548325.1 (*F.cul* F: CACCGTCATTGGTATGTTGTCACT, *F.cul* R: CGGGAGCGTCTGATAGTCG) and for pepper gDNA GenBank accession XM_016716654.2 (*Act* F: GGACTCTGGTGATGGTGTCAGC, *Act* R: GTCCCTGACAATTTCCCGCTCAG) was used. For *B. cinerea* (*B.cin* F: ATTCCACAATATGGCATGAAATC, *B.cin* R: ATGTTATCTCATGTTATCTC) the primer sequences were adapted from the study of Zhang et al. (2013) [33]. For the qPCR, a PowerUp™ SYBR™ Green Master Mix (Applied Biosystems, Thermo Fisher Scientific, Vilnius, Lithuania) was used with a standard cycling with the annealing set to 60 °C in a real-time PCR thermal cycler qTOWERiris (Analytik Jena, Jena, Germany).

### 2.3. Analytical Measurements

#### 2.3.1. Sample Preparation for Analytical Measurements

Infected peppers were compared to their mock-infected counterparts in terms of their total polyphenolic content (TPC) and antioxidant capacity (FRAP). For the sample preparation the homogenized freeze-dried samples were used. For the extractions, 60:39:1% *v*/*v* methanol/distilled water/formic acid was mixed with freeze-dried pepper powder.

#### 2.3.2. Total Polyphenolic Content (TPC) and Antioxidant Capacity (FRAP) Measurements

The TPC was measured according to Singleton and Rossi (1965), at λ = 760 nm with a Hitachi U-2900 spectrophotometer in three repetitions [43]. TPC was calculated based on the calibration curve of 0, 6, 12, 18, 24 and 30 µg/mL gallic acid, and the results are expressed as mg gallic acid equivalent (Ga)/g dry weight. FRAP was measured following Benzie and Strain (1996), at λ = 593 nm with a Hitachi U-2900 spectrophotometer in triplicates [44]. The results are expressed in µmol ascorbic acid equivalent (As)/g dry weight, calculated against the calibration curve of 0, 6, 12, 18, 24 and 30 µmol/l ascorbic acid.

#### 2.3.3. HPLC Determination of Phenolics and Vitamin C

The evaluation of the Vitamin C, anthocyanin and polyphenolic composition of the samples was performed by a high-performance liquid chromatography (HPLC)-diode-array detector (DAD) injection to identify changes in the compounds in each color group and each phenophase upon infection by *B. cinerea*. The extraction of the samples was performed by mixing 0.5 g of freeze-dried pepper samples with 25 mL of 70:30 *v*/*v*% 3% metaphosphoric acid/methanol. The mixture was shaken for 15 min at 300 rpm followed by an ultrasound bath for 5 min, centrifugation at 5000× *g* for 5 min, then filtration through a 0.22 µm pore sized syringe filter into glass vials.

For the measurements, an HPLC system (Hitachi Chromaster, Tokyo, Japan) with the following column type was used, Supelco Ascentis^®^ Express 90 Å C18-PCP (St. Louis, MO, USA) 15 cm × 4.6 mm, 2.7 µm, with gradient elution of 1% ortho-phosphoric acid (A) and acetonitrile (B). The gradient elution started with 1% B in A, changed to 20% B in 20 min, remained isocratic for 10 min, changed to 30% B in 5 min, remained isocratic for 10 min, and turned to 1% B in 5 min. The DAD detection was between 190 nm and 700 nm. The operation and the data processing were performed using EZChrome Elite software (ver. 3.3.2). The quantification was based on recording the area at the maximum absorbance wavelength of each compound and relating it to the standard solutions acquired by Sigma-Aldrich via Merk (Budapest, Hungary). In those cases when a standard was not available, the compounds were putatively identified based on their spectral characteristics, chromatographic behavior and the literature data. The results are expressed as μg/g dry weight. For the measurements, four biological replicates were applied.

### 2.4. Statistical Analysis

For the descriptive statistics we used the IBM SPSS Statistics version 29.0.1.0 software. Analysis of variance (one way or multiple) was applied to assess the statistical significance between the different color and maturity groups in terms of pathogen growth and inoculation tests, as well as total polyphenolic content and total antioxidant capacity measurements. Where a statistically significant effect was found (*p*-value < 0.05), Tukey’s test or Wald Χ^2^ test were further used.

Further analyses were conducted with the open source statistical program R (version 4.5.0). Data import and cleaning were handled with readxl, dplyr, tidyr and tibble. Statistical computations relied on base R together with dplyr; compact display of multiple-comparison results used multcompView. General plots were produced with ggplot2; figure alignment used cowplot, patchwork and grid packages. Heatmap was generated with ComplexHeatmap, with color mapping and track annotations supported by the circlize package. Image post-processing for figure assembly used png and magick packages.

## 3. Results and Discussion

### 3.1. Pathogen Growth Tests In Vitro

**Figure 1:**
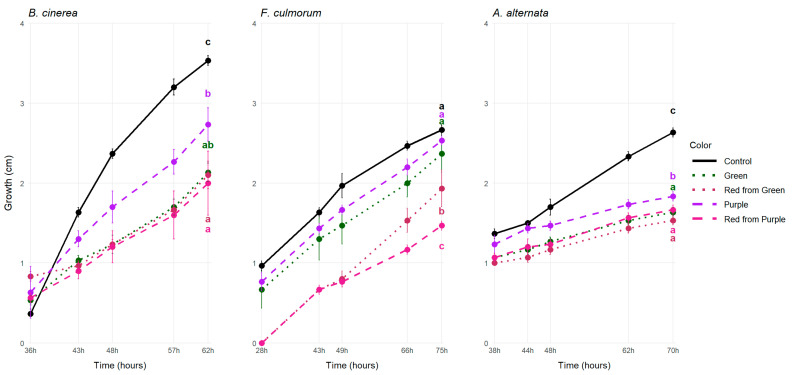
Mean colony growth (in cm) on PDA supplemented with 50% sterile pepper juice at the last sampling date. The different lower case letters with matching colors mean significant statistical differences (*p*-value < 0.05) among the control (plain PDA), purple, green, red from green and red from purple pepper juices in three replicates.

Anthocyanins have been associated with reduced susceptibility to *B. cinerea* in the case of grapes [45] and tomatoes [32,33]. To test the hypothesis that anthocyanins may exert a protective role against storage pathogens *B. cinerea*, *F. culmorum* and *A. alternata* in the case of pepper, these pathogens were grown on a PDA medium supplemented with the economically ripe green and purple pepper juice and on a media supplemented with their biologically ripe red counterparts (Figure 1).

Interestingly, fungal growth was the strongest on the purple pepper juice-supplemented media after the control, and the least amount of growth was measured in the case of the biologically ripe pepper juices-supplemented media (Figure 1). In the case of *Botrytis*, the growth on the purple media was 26% greater compared to the red from green one, while in the case of the *Fusarium* it was 23% greater, and this difference was the smallest in the case of *Alternaria*, where the growth on the P media was only 13% greater than on the RG media (Figure 1).

Overall, addition of the filtrates to the media did hinder the growth of the pathogens compared to the control. This effect was significant in the case of *B. cinerea* and *A. alternata*, indicating the presence of molecules that are active against the fungal pathogens in vitro as well, although we could not detect strong antifungal activity in the anthocyanin-rich media.

This aligns with Zhang et al.’s (2013) study, where *B. cinerea* growth was not inhibited on either the red or the purple tomato juice-supplemented media. Hence, they concluded that resistance against the pathogen requires living cells [33]. However, one in vitro study indicated that addition of anthocyanins cyanidin-3-glucoside or pelargonidin-3-glucoside to PDA media could effectively reduce the growth of *B. cinerea* in higher concentrations [31]. Therefore, we hypothesize that the enrichment of the media with the anthocyanin-rich pepper filtrate may not have reached the minimum inhibitory concentration. Similarly to our case, where only the purple extract-supplemented media differed, when *Alternaria* was grown on immature green and red ripe pepper extract-supplemented media, there was no significant difference in pathogen growth in between the fruit colors [46].

### 3.2. Artificial Infection Studies In Vivo with B. cinerea, F. culmorum and A. alternata

**Figure 2:**
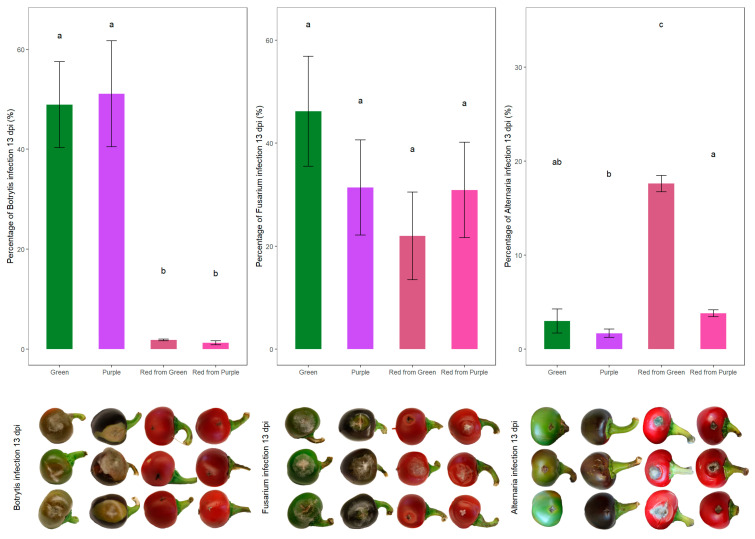
Percentage of infection of *B. cinerea*, *F. culmorum* and *A. alternata* after 13 days post-inoculation (dpi) of infected plants from each color group (n = 10); different lower case letters mean significant statistical differences (*p*-value < 0.05). The lower images display the most common phenotype of infection appearing at 13 dpi.

Although in vitro assay provided a controlled environment to test the antifungal activity of fruit extracts, it did not allow for the study of active host responses occurring in whole fruits. Therefore, in vivo artificial infection experiments were conducted in parallel to the in vitro assays to further evaluate the effectiveness of the NILs against storage pathogens (Figure 2).

**Figure 3:**
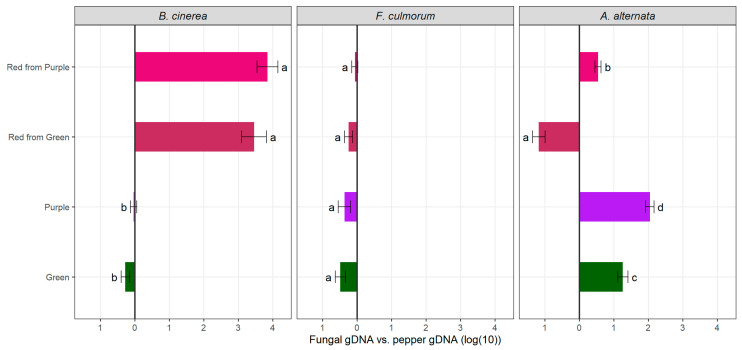
Quantitative PCR at 13 dpi of each color group (n = 3); fungal growth was calculated by comparing the ratio of *Botrytis*, *Fusarium* or *Alternaria* DNA to pepper DNA. Different lower case letters mean significant statistical differences (*p*-value < 0.05) between the groups, bars to the left represent fungal gDNA excess and bars to the right represent pepper gDNA excess.

For *B. cinerea*, the infection phenotype yielded unexpected results (Figure 2). On the ER fruits—green and purple—severe symptoms of gray mold developed around the site of the inoculation, on the other hand, the infection was less severe on the BR red counterparts. The average infection percentage of the economically ripe fruits was 50.03%, as opposed to 1.54% in the biologically ripe fruits. In our case, necrotic lesions at the site of infection, even at 13 dpi, were characteristic only of the BR red pepper fruits (Figure 2). We could not conclude the beneficial effects of anthocyanins against the pathogen infection, as qPCR with the DNA extracted from the infected fruits at 13 dpi also confirmed that there was significantly less *Botrytis* growth on the RP and RG fruits than on the G and P economically ripe fruits (Figure 3).

Infection carried out with *Fusarium* yielded uniform results across color groups and phenophases, by both phenotypic evaluation and qPCR (Figure 2 and Figure 3). Although anthocyanins have been hypothesized to act against *Fusarium* infections, in our case the averages of infection percentages did not confirm this hypothesis; G: 46.21%, P: 31.37%, RG: 22.04% and RP: 30.86% (Figure 2).

Inoculating the NILs with *A. alternata* resulted in more severe infections at the biologically ripe phenophase; however, based on the phenotypic evaluation of disease severity, the green-colored economically ripe fruits’ infection rate did not differ significantly from the ripe RP fruits’ infection rate (Figure 2). The average infection percentages at 13 dpi were G: 2.98% and P: 1.68%; the average of the economically ripe fruits: 2.33%, RG: 17.59% and RP: 3.80%; and the average of the biologically ripe ones: 10.69% (Figure 2). Although the NILs used in this study share a common genetic background, they differ in their anthocyanin content, resulting in a somewhat distinct biochemical composition; therefore, it is also interesting to compare the averages of the two NILs; the purple ones yielded an infection percentage of 2.75%, whereas the green ones yielded an infection percentage of 10.28%.

According to the generalized linear model (Gamma with log link), there was a significant effect of the variation sources: genotype (Gt), phenophase (Pp) and the interaction of Gt × Pp (Table 1).

For *Botrytis*, infection severity was primarily influenced by phenophase (Χ^2^ = 1185.514, *p* < 0.001), whereas genotype alone had no significant effect (Χ^2^ = 2.457, *p* < 0.117); however, the genotype × phenophase interaction showed a weaker but statistically significant impact (Χ^2^ = 4.028, *p* < 0.045).

In the case of *Fusarium*, no significant differences were attributable to genotype alone, while both phenophase (Χ^2^ = 7.098, *p* < 0.008) and the genotype × phenophase interaction (Χ^2^ = 6.496, *p* < 0.011) exerted significant effects on disease intensity. These tendencies can also be seen in Figure 2—where the economically ripe fruits’ average infection percentage was 38.79%, whereas the biologically ripe fruits’ was 26.45%—and can be statistically proven by the X^2^ test (Table 1).

For *Alternaria,* infection severity was significantly affected by genotype (Χ^2^ = 101.634, *p* < 0.001), phenophase (Χ^2^ = 154.047, *p* < 0.001) and their interaction (Χ^2^ = 21.218, *p* < 0.001), indicating that both main factors and their combined effect played a decisive role (Table 1).

The ripening-dependent susceptibility of tomatoes to *B. cinerea* is well-established. This increased susceptibility has been linked to ripening regulatory pathways, such as the NOR transcription factor [28]. Further, transcriptomic and proteomic studies have revealed that defense-related proteins are less abundant in mature green (MG) fruits than in red ripe (RR) fruits [47,48]. Moreover, *B. cinerea* can accelerate host ripening, thereby facilitating colonization [49]. Upon infection with *B. cinerea* and *F. acuminatum*, the pathogens were able to grow on the surface of MG tomato fruits, but only the RR fruits showed symptoms of rot [47,50]. When MG tomato fruits were inoculated with *B. cinerea* at 1 dpi, the necrotic lesions were limited to the site of infection; however, RR fruits started to develop tissue rot and fungal growth. This localized necrosis could be attributed to oxidative burst, as hydrogen peroxide accumulation was detected 3–4 cell layers deep around the infection site [28]. Differential expression studies revealed that during maturing from MG to RR, the expression of defense-related genes undergoes changes; for example, the expression of genes that are involved in the mediation of reactive oxygen species (ROS) levels declines. This reduced expression could lead to a reduction in preformed defense as a result of losing control of ROS levels [47]. Our results contradict studies carried out with purple tomatoes, where anthocyanin accumulation has been directly linked to extended shelf life and enhanced resistance to *B. cinerea*. Anthocyanin-rich tomato fruit exhibited delayed overripening and reduced susceptibility to gray mold [32], and a further study reported that anthocyanin-rich lines displayed a doubled shelf life compared to the wild-type tomatoes, as anthocyanins added to the overall antioxidant capacity, therefore contributing to the reduction in oxidative stress [33].

According to Le et al. (2013), who studied postharvest *B. cinerea* infection in pepper cultivars at different ripening stages, in the cultivar ‘Papri Queen’, the strongest symptoms occurred at the breaker red stage, whereas in the ‘Aries’ cultivar, the fully ripe fruits developed disease symptoms more rapidly [51]. This indicates that in pepper, infection severity is not strictly correlated with ripening stage but is also strongly influenced by genotype [52]. Additionally, peppers infected with *Colletotrichum* sp. also exhibited genotype and ripening stage-dependent differences in pepper fruit responses, with unripe fruits showing larger lesion diameters on average [53]. Consistent with this, our observation of higher infection rates in the economically ripe stage compared with the biologically ripe stage likely reflects genotype–phenophase interactions rather than a simple ripening-dependent trend.

Effects of anthocyanins on the growth and mycotoxin production of *F. culmorum* was studied by Trávníčková et al. (2024) in the case of differently colored wheat grains. Red grains showed the lowest deoxynivalenol (DON) content; however, this was not consistent across all crop years, while grains with purple pericarp exhibited a constant and moderate level of DON across all studied crop years [54]. This was also confirmed by Gozzi et al. (2023), who found that blue wheat grains—anthocyanins accumulating only in the aleuron—exhibited higher susceptibility to *Fusarium* than those where the anthocyanin pigmentation was characteristic to the pericarp [55]. Further, when the effect of anthocyanidins was studied on the conidial growth on *Fusarium avenaceum*, cyanidin 3-O-glucoside and delphinidin 3-O-glucoside were found to significantly reduce conidial germination [56]. Therefore, it was hypothesized that the different accumulation pattern of these pigments might act against the early stages of infection; however, in our study, this phenomenon was not observed, although peppers have also been found to accumulate delphinidins in their pericarp [57]. Anthocyanin accumulation in peppers has also been studied in relation to *Phytophthora capsici* infection. When the *CaMYB* gene—a key factor in the anthocyanin biosynthesis of pepper—was silenced, symptoms of *P. capsici* infection were quicker to develop on the silenced plants’ leaves than on the control, indicating that anthocyanins might be functioning as a first line of defense [34].

The ripening-dependent manner of *Alternaria* infection was also studied in tomatoes; upon comparing MG and RR phenophases of three varieties kept at 25 °C for 10 days, the RR fruits showed the quickest progression of disease; on the contrary, the MG stage of ‘Charleston’ and ‘Geronimo’ varieties showed no lesions during the storage period [58]. The phenophase dependency of *A. alternata* infection in pepper fruits has been described previously; no lesions were evident after 10 dpi in the mature green fruits; however, with as little as 10% red coloration on the fruits, lesions were formed [24]. The ripening stage dependency of *Alternaria* infection in peppers was also studied in a temperature-dependent manner [46]. In cold storage, the symptoms and lesion diameter were similar in the economically and biologically ripe fruits; however, at 22 °C lesions were significantly larger on the ripe fruits [46]. This is in line with our study, as the symptoms of the fruits kept at 25 °C were more advanced on the red ripe fruits, while only smaller lesions were observable on the G and P fruits (Figure 2). This was confirmed by qPCR analyses as well, where we detected higher fungal load in the biologically ripe red peppers, with the RG exhibiting the most fungal DNA compared to the pepper DNA, which also aligns with the phenotypic evaluation of the disease severity (Figure 2 and Figure 3).

### 3.3. Total Polyphenolic Content, Antioxidant Capacity and Anthocyanin Content of the Infected Pepper Fruits

As the differences could be attributable to the metabolic changes occurring during ripening, the total polyphenolic content and total antioxidant capacity were measured by spectrophotometric methods and by HPLC, together with the total monomeric anthocyanin content. Measurements were carried out at the time of harvest (Week 0) and at 13 days post infection with the three pathogens, respectively, and mock-infected plants were used for the control (Table 2).

Both with the spectrophotometric methods and with the HPLC, a similar trend was visible in terms of changes in FRAP, TMA and TPC values (Table 2). At the time of the harvest, the P fruits showed the highest values for all the tested parameters; TPC: 610.69 ± 54.16 mg GA/g DW, which showed a statistically significant difference from the G, RG and RP samples. As for the FRAP of P samples: 293.76 ± 27.50 µmol As/g DW differed significantly from the rest of the samples, and the same trend was also observed for the HPLC measurement, where the total polyphenolics measured was higher and the difference compared to the rest of the samples was statistically significant. At Week 0, statistically significant differences were also scored between the phenophases; in the case of FRAP, in the economically ripe fruits, we measured 256.76 ± 1.66 compared to the biologically ripe 189.07 ± 9.59 µmol As/g DW. There was a significant decrease as well in the TPC values when measured by HPLC, although when spectroscopy was applied, the decrease in the ripened fruits was only trend-like. The TPC measured using a spectrophotometric method showed a greater decrease in the purple samples compared to the green ones, whereas this was the contrary when TPC was measured using the HPLC method; nevertheless, there was a decrease in the TPC from Week 0 to the control stage (mock-infected plants stored at 25 °C for 13 days) (Table 2). The 13-day storage period also affected the anthocyanin levels; however, the decrease was not statistically significant (Table 2). FRAP values changed according to the phenophase studied; the economically ripe fruits showed a significant decrease, whereas a trend-like increase was detected in the biologically ripe fruits (Table 2).

In the case of the infections with the three storage pathogens, FRAP, TMA and TPC values all differed significantly from the Week 0 data, and most of them showed significant differences compared to the control data as well. There were only two cases where there was only a trend visible; TMA of the RP in the case of the control (17.81 ± 4.81 µg/g DW) did not differ from the *Botrytis*-infected fruits (2.85 ± 0.71 µg/g DW) and the TPC measured by HPLC in the case of the control RG fruits (468.98 ± 34.65 µg/g DW) was not significantly different from that of the *Botrytis*-infected fruits (326.88 ± 11.25 µg/g DW), although they differed from the Week 0 data (Table 2.).

The correlation analysis showed high association between the phenophase and the infection percentage of the *Alternaria* (r = 0.646), where the biologically ripe phenophase showed more fungal growth than the economically ripe fruits (Figure 2, Table 3). The ripening showed a negative correlation with FRAP (r = −0.200), meaning that the biologically ripe fruits possessed lower antioxidant capacity (Table 2 and Table 3). The FRAP and TPC values showed a strong positive association in each case studied (r = 0.830, r = 0.835, r = 0.834, in the case of the *Alternaria*, *Fusarium* and *Botrytis*, respectively). The infection percentage showed a negative correlation with the genotype (r = −0.389), indicating that the green NIL was more prone to *Alternaria* infection (Figure 2, Table 3).

Regarding the *Fusarium* data, a negative correlation was scored between the phenophase and infection percentage (r = −0.531). As Figure 2 shows, the infection was more severe in the case of the economically ripe fruits on average. The TPC and FRAP values showed a positive association with the genotype, indicating that the purple NIL exhibited higher scores for these measured indices (Table 2 and Table 3).

In the case of the *Botrytis* data, a negative correlation was observed between phenophase and infection severity (r = −0.974), with economically ripe fruits developing greater fungal growth. TPC (r = −0.597) and TMA (r = −0.462) measured by HPLC showed a negative correlation with the phenophase, indicating that the biologically ripe fruits contain smaller amounts of these compounds. The genotype studied showed a strong positive correlation with the measured variables, TPC (r = 0.208), FRAP (r = 0.202), HPLC–TPC (r = 0.526) and HPLC–TMA (r = 0.519), indicating that these compounds are more abundant in the purple NIL. The two different approaches for the TPC measurements also showed a strong correlation (r = 0.453), and the amount of anthocyanins measured also correlated with the measured polyphenolics (r = 0.462 measured by spectrophotometer, r = 0.858 measured by HPLC) and with the antioxidant capacity (r = 0.458). Although TMA showed a strong positive correlation with the infection percentage of *Botrytis* (r = 0.486), as Figure 2 shows, the purple NIL was the most severely infected fruit (Figure 2, Table 3).

Our results align with Marin et al. (2004), who also observed decreasing polyphenolic content during ripening [59]. As for the differences between the Week 0 and control data, Garra et al. (2020) found that the amount of polyphenols during storage is affected by genotype, as in some accessions there was a 32% increase, while others showed a 22–24% decrease during storage [60]. When infecting pepper cultivars with *Alternaria*, Tukuljac et al. (2023) obtained genotype-dependent responses in terms of TPC and antioxidant capacity, with ‘Amfora’ and ‘Una’ showing an increase, whereas in ‘Kurtovska kapia’ TPC remained unchanged and the antioxidant capacity showed a decrease upon infection [61]. Upon investigating the effect of *Alternaria* on the antioxidant responses of tomato, Meena et al. (2017) found a significant decrease in the TPC with the dpi [62]. A study conducted by Tzortzakis (2019) found that FRAP showed an increase, while the total phenolics did not showing significant differences between the *Botrytis*-infected tomatoes and the control [63]. These differences may arise from the different experimental settings in terms of storage time, plant material or pathogen strain used. For example, in terms of storage time, as Bui et al. (2019) concludes, Vitamin C levels of infected apples were comparable to the control after 5 days, but showed a decrease after 14 days of incubation [64].

### 3.4. HPLC Determination of Phenolics and Vitamin C of the Botrytis-Infected Fruits

As *Botrytis* infection showed contradicting results with the purple tomato studies, subsequent biochemical analyses focused on this pathogen. To gain a deeper understanding of the changes in polyphenolics upon *Botrytis* infection, HPLC measurement was carried out (Table 4 and Table 5).

**Figure 4:**
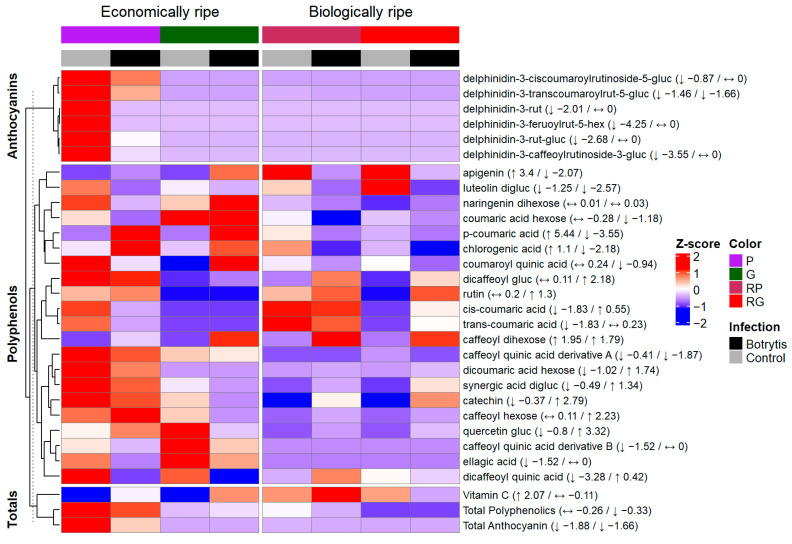
Heatmap (Z-score) representing the differences in the amount of the different compounds between the *Botrytis*-infected (black) and the mock-infected (gray) control group; purple color (P) represents the economically ripe purple NIL, green color (G) represents the data of the economically ripe green NIL, maroon (RP) represents the Red from Purple biologically ripe stage of the purple NIL, red (RG) represents the Red from Green biologically ripe stage of the green NIL and arrows and numbers in the brackets indicate the direction of the fold of change in the Z-score related to the metabolites measured in the economically ripe stage or at the biologically ripe stage. hex: hexoside, rut: rutinoside, gluc: glucoside.

The changes in the metabolite levels were analyzed by pairwise column mean comparisons using two-sample *t*-tests (two-sided, equal variances assumed), with a significance level of *p* < 0.05. Three aggregated values, Vitamin C, total polyphenolics and total anthocyanins, were analyzed. We identified a phenophase-dependent change in the case of Vitamin C: in the G and P fruits the content of it increased significantly compared to the control, whereas in the case of RP no significant change was detected, and in the RG fruits, a significant decrease was found (Table 4 and Table 5). This ripening stage difference in the Vitamin C content may arise from the 13-day-long storage at 25 °C and from the *Botrytis* infection itself as well, as *Botrytis* accelerates ripening processes, and with ripening the Vitamin C content increases (Table 4 and Table 5). Meanwhile, in the RG fruit, a strong decrease was experienced as expected upon oxidative stress. In terms of the anthocyanin content, a strong decrease in the total amount of anthocyanins was measured in both phenophases of the NILs (−1.88 and −1.66) (Figure 4). A similar reduction was also experienced in the individual anthocyanin compounds (Figure 4). We observed a severe degradation—a 73% reduction compared to the control in the case of the P fruits—of these compounds (Table 4, Figure 4), which can be explained by the oxidative degradation of these metabolites by the pathogen.

Ascorbic acid is one of the most well-known and major antioxidant molecules of peppers, acting as a key substrate for neutralizing reactive oxygen species [65]. A decrease in the amount of it upon pathogen infection has already been demonstrated in studies involving strawberry [66], *Arabidopsis* [67] and apple [64]. As Meena et al. (2017) reports, ascorbic acid was also depleted after *A. alternata* infection [62], and this depletion was also confirmed in the case of peppers in a genotype-dependent manner [61]. Studying the polyphenolics, Zimdars et al. (2017) found that besides caffeic and ferulic acids, malvidin-3-O-glycosides are also oxidized rapidly by the secretomes of the pathogen [68]. On the other hand, when white-skinned berries were analyzed after *Botrytis* infection, Blanco-Ulate et al. (2015) found that the anthocyanin accumulation was induced, especially in terms of the production of cyanidin and delphinidin glycosides [69]. This is unlike Echnenique-Martínez et al.’s (2023) study, where upon infecting the strawberries with *Botrytis* there was a significant reduction in the amount of pelargonidin-3-glucoside [66]. A study carried out on *Botrytis*-infected grape berries showed that upon infection, the amount of anthocyanins in the skin decreased by more than 80% [70]. Gimenez et al. (2023) studied the effect of active laccase extracts obtained from a *Botrytis* isolate on different anthocyanins and found that petunidin-3-O-glucoside and delphinidin-3-O-glucoside were the fastest to be degraded by the enzyme [71]. In our case, in the P and RP fruits, the most prevalent anthocyanin was the delphinidin-3-transcoumaroylrutinoside-5-glucoside; the amount of this compound in the infected fruits decreased by approximately 70% compared to Week 0, and by approximately 63% compared to the control fruits in the P fruits (Table 4, Figure 4).

An overall decreasing trend was observed in the aggregated TPC value; however, some metabolites showed an increase upon infection, either at both phenophases or in a single phenophase (Figure 4). For example, amongst the simple hydroxycinnamic acid derivatives, coumaric acids showed different responses to the infection according to the phenophase studied. The greatest difference was recorded in the case of p-coumaric acid, with higher values measured in the economically ripe phenophase. As for the more complex chlorogenic-type derivatives, there was a also difference recorded between the phenophases in the response of chlorogenic acid levels to the pathogen infection. Other flavonoids, such as the luteolin diglucoside, showed a decrease, while the levels of rutin remained unchanged in the P and RP fruits.

In the case of the polyphenolics, the trend is not so clear; for instance, Muñoz Aries et al. (2021) detected higher amounts of polyphenols in the inoculated *Rubus glaucus* fruits, and the amounts of them correlated with the *Botrytis* infection severity [72]. However, when the amount of polyphenolics was studied in relation to the days post-inoculation, Wang et al. (2012) found a significant decrease in TPC in each strawberry genotype studied [73].

### 3.5. Relationship Between Botrytis Infection Severity and Metabolite Levels

**Figure 5:**
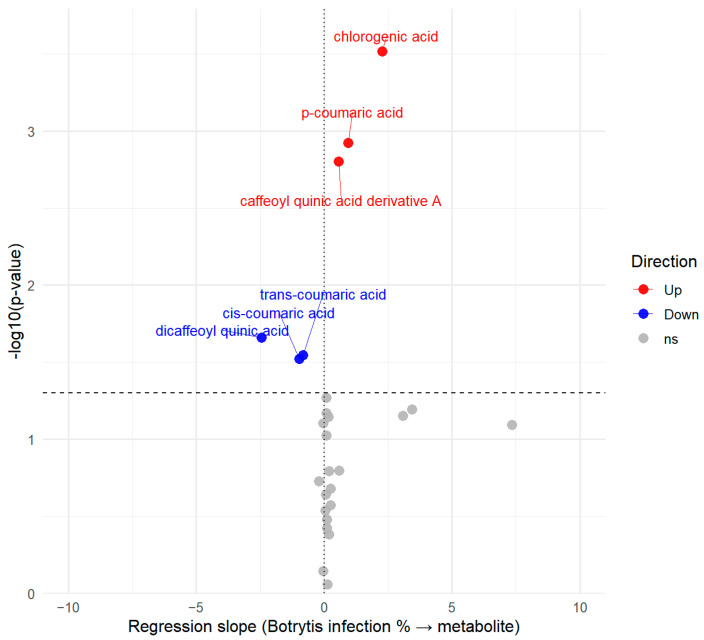
Volcano plot representing the relationship between the *Botrytis* infection and the metabolites measured, with red metabolites showing positive correlation, blue metabolites showing negative correlation with the infection percentage and gray color showing a non-significant (ns) relationship.

Since infection severity was also evaluated, it was possible to examine the relationship between the level of infection and the abundance of individual compounds of the *Botrytis*-infected batch. This relationship is illustrated in a volcano plot (Figure 5).

Compounds that did not show a significant association with infection severity are located underneath the vertical threshold line displayed in gray. The volcano plot highlights several phenolic acid compounds located above the significance threshold line that were modulated by the *Botrytis* infection. Chlorogenic acid, p-coumaric acid and a caffeoyl quinic acid derivative increased with infection (positive slopes in red), whereas cis- and trans-coumaric acid and dicaffeoyl quinic acid decreased (negative slopes indicated with blue). For metabolites exhibiting statistical significance, the magnitude of their deviation was also taken into account, as reflected by the distance from the central vertical line in the volcano plot.

To enable a more detailed assessment of the amount of metabolites across the infection levels, the samples were further grouped into three categories according to *Botrytis* infection percentages: low (0–15%), moderate (15–45%) and severe (>45%). Therefore, the influence of *Botrytis* infection on the metabolic composition was evaluated using multivariate analysis of variance (MANOVA), allowing for parallel analysis of multiple metabolites. The grouping of metabolites was similar to the one used for the creation of the heatmap: the totals and the anthocyanins were grouped. The polyphenolics were, however, separated into three blocks: simple hydroxycinnamic acid derivates, more complex chlorogenic-type derivatives and other flavonoids.

MANOVA showed that the *Botrytis* infection had no significant effect (Wilks’ Lambda = 0.293, F(6,12) = 1.693, *p* = 0.206) on the totals (Vitamin C, total anthocyanin and total polyphenolics). Upon focusing on each component separately, no significant differences were scored either, although the total polyphenolics showed a trend-like pattern with regard to the infection groups (*p* = 0.061). Analyzing the delphinidin derivatives group (Wilk’s Lambda = 0.229, F(8,10) = 1.360, *p* = 0.318) showed that there was no significant effect on this group, although the amount of these compounds decreased significantly upon infection (Table 2 and Table 4, Figure 4). However, this effect was not significant compared to the three infection categories, meaning that *Botrytis* infection, regardless of severity, leads to the degradation of these compounds. Testing the hydroxycinnamic acid derivatives, the severity of *Botrytis* infection was not significant in this group of metabolites (Wilks’ Lambda = 0.006, F(4,2) = 5.951, *p* = 0.149). However, the overall effect was not significant. When analyzed separately, several compounds showed trend-like differences between infection groups: caffeoyl quinic acid derivative A (*p* = 0.057), caffeoyl quinic acid derivative B (*p* = 0.108), p-coumaric acid (*p* = 0.069), cis-coumaric acid (*p* = 0.136) and trans-coumaric acid (*p* = 0.106). MANOVA indicated a significant overall effect (Wilk’s Lambda = 0.000, F(8,2) = 26.230, *p* = 0.37) of infection groups on the chlorogenic-type derivatives. In our study, the amount of CGA significantly increased in the P and G fruits upon infection, but showed a decrease in the biologically ripe PR and PG fruits (Figure 4). Further, in the univariate tests, chlorogenic acid (*p* < 0.001) and dicaffeoyl-glucoside (*p* = 0.002) were significantly affected by the infection groups, and there was also a trend visible for the dicaffeoyl quinic acid (*p* = 0.077). As for the rest of the compounds, MANOVA showed no significant overall effect (Wilks’ Lambda = 0.000, F(6,2) = 18,421, *p* = 0.052). When tested separately, quercetin glucoside was greatly affected by the infection groups (*p* < 0.001). In the case of rutin, there was a trend-like manner (*p* = 0.055); however, the other metabolites, catechin, naringenin dihexose and apigenin, as well as the luteolin diglucoside, were not significantly affected by the infection groups. These patterns indicate that infection-induced changes in hydroxycinnamic acids may be more relevant for postharvest defense in pepper than changes in bulk anthocyanin levels.

Some of these compounds have already been proven not only to be responsive, but also to be effective against infection; for example, Liu et al. (2020) found that p-coumaric acid significantly inhibited the mycelia growth of *Botrytis* in sweet cherries [74]. Glazener (1982) found an increase in this compound after inoculating young tomato fruits with *Botrytis* [75]. Chlorogenic acids (CGAs) are well-known to inhibit spore formation and reduce the fungal growth of *F. solani* and *B. cinerea* [76] by disrupting the cell membrane [77]. López-Gresa et al. (2011) found that upon infecting tomatoes with *Pseudomonas syringae*, there was an accumulation in CGAs [78]. Exogenous application of quercetin and cathecin hindered the germ tube and mycelia growth of *Botrytis* [31]. However, in our case, quercetin was greatly affected by the infection groups. This could be explained by the laccases produced by *Botrytis*; as Quijada-Morin et al. (2018) found, these laccases and peroxidases can detoxify or degrade these hydroxycinnamates. In their study, they determined that the best substrate was quercetin [79]. On the other hand, when rutin was tested against *Alternaria*, *F. solani* and *B. cinerea*, it was found that it was only effective against the growth of *B. cinerea*, as it stimulated the formation of conidia in the case of *Alternaria* and *F. solani* [80].

Overall, this study combines phenotypic, spectrophotometric and targeted metabolomic analyses to assess how different pigment composition, phenophase and genotype affect the responses of pepper fruits to major postharvest fungal pathogens. However, there are several limitations of this work, i.e., the experiments were performed under controlled laboratory conditions using artificial inoculation and a single storage temperature, which may not fully represent the variable environments of postharvest handling and distribution. Further, only *C. annuum* var. *cerasiforme* NILs were used, and responses may differ in other varieties. Moreover, the strains were not isolated from the used NILs, which may slightly limit the representativeness of the infection dynamics observed. Detailed metabolite profiling was carried out only for *B. cinerea*, while *A. alternata* and *F. culmorum* were examined primarily at the phenotypic level. Future studies should extend metabolomic analyses to other pathogens, storage periods or temperatures, as well as genotypes. It is also important to integrate transcriptomic analysis to better link metabolic changes with gene expression. Functional validation of key metabolites—such as chlorogenic and p-coumaric acids—in antifungal defense will be essential to clarify their exact roles so that they can serve as biochemical markers for breeding programs targeting improved postharvest disease tolerance in peppers. These findings may help breeding efforts aimed at combining pigmentation and nutritional value with improved resistance against storage pathogens, thus supporting a more sustainable postharvest management strategy that relies on natural, polyphenol-based defenses rather than synthetic fungicides.

## 4. Conclusions

In conclusion, our study demonstrates that pepper susceptibility to postharvest pathogens is shaped primarily by ripening stage and genotype rather than by anthocyanin accumulation alone, in contrast to findings in tomato studies. Economically ripe fruits were more vulnerable to *Botrytis cinerea*, while biologically ripe fruits showed higher susceptibility to *Alternaria alternata*. *Fusarium* infections were not strongly influenced by pigmentation. *Botrytis* infection led to a marked depletion of total phenolics, antioxidant capacity, Vitamin C and anthocyanins. Multivariate analysis revealed that *Botrytis* infection percentage did not significantly alter the totals of Vitamin C, total anthocyanins and total polyphenols, indicating that their levels are altered by the infection itself and not by the severity of it, yet several individual metabolites showed correlation with infection severity. Chlorogenic acid and certain hydroxycinnamic acid derivatives (caffeoyl quinic acids, coumaric acids) and flavonoids such as quercetin-glucoside and rutin were affected by the severity of *Botrytis* infection. Total anthocyanin content and the delphinidin derivatives measured decreased upon infection; however, this effect was not significant in correlation with infection severity, indicating that *Botrytis* infection in a low, moderate or severe amount will lead to the degradation of these compounds. Therefore, although anthocyanins have been proven to be effective against *B. cinerea* infection in the case of purple tomatoes, we could not conclude the beneficial effects of these molecules against this pathogen in the case of pepper.

## Figures and Tables

**Table 1:** Infection percentages of the three storage pathogens analyzed using a generalized linear model; fixed factors were the genotype (Gt), phenophase (Pp) and their interaction (Gt × Pp).

Pathogen	Source of Variation	Wald Χ^2^	*p*-Value
*Botrytis*	Genotype (Gt)	2.457	<0.117
	Phenophase (Pp)	1185.514	<0.001
	Gt × Pp	4.028	<0.045
*Fusarium*	Genotype (Gt)	0.031	<0.860
	Phenophase (Pp)	7.098	<0.008
	Gt × Pp	6.496	<0.011
*Alternaria*	Genotype (Gt)	101.634	<0.001
	Phenophase (Pp)	154.047	<0.001
	Gt × Pp	21.218	<0.001

**Table 2:** Antioxidant capacity (FRAP), anthocyanin content (TMA) and total polyphenolic content (TPC) of the samples measured by spectrophotometry and HPLC at the time of harvest (Week 0) and at 13 days post infection.

		FRAP [µmol As/g DW]	TMA–HPLC [µg/g DW]	TPC [mg GA/g DW]	TPC–HPLC [µg/g DW]
Purple	Week 0.	293.76 ± 27.50 a	1027.08 ± 52.23 a	610.69 ± 54.16 a	2654.90 ± 121.28 a
	Control	234.86 ± 21.48 b	905.64 ± 115.93 a	354.54 ± 48.89 b	2518.99 ± 113.90 a
	*Botrytis*	77.84 ± 3.05 c	271.26 ± 71.39 b	53.51 ± 1.97 c	1814.64 ± 29.71 b
	*Fusarium*	84.57 ± 4.03 c		42.21 ± 4.80 c	
	*Alternaria*	88.34 ± 3.47 c		58.53 ± 2.36 c	
Red from Purple	Week 0.	211.65 ± 15.34 a	105.82 ± 25.26 a	467.66 ± 57.27 a	1036.99 ± 21.69 a
	Control	214.83 ± 15.51 a	17.81 ± 4.81 b	266.30 ± 61.29 b	1017.69 ± 53.12 a
	*Botrytis*	73.17 ± 5.92 b	2.85 ± 0.71 b	47.04 ± 5.11 c	732.03 ± 7.17 b
	*Fusarium*	91.59 ± 3.59 b		47.61 ± 3.49 c	
	*Alternaria*	89.99 ± 4.01 b		55.80 ± 3.32 c	
Green	Week 0.	219.77 ± 15.07 a	nd.	328.20 ± 18.60 a	1401.22 ± 36.56 a
	Control	199.10 ± 8.97 a	nd.	273.14 ± 19.39 b	784.42 ± 13.93 b
	*Botrytis*	59.71 ± 10.65 c	nd.	44.64 ± 5.44 c	911.28 ± 8.62 c
	*Fusarium*	88.76 ± 5.66 b		51.20 ± 6.52 c	
	*Alternaria*	90.39 ± 5.81 b		67.87 ± 2.70 c	
Red from Green	Week 0.	166.50 ± 5.30 a	nd.	292.76 ± 40.29 a	1264.37 ± 87.42 a
	Control	173.90 ± 12.14 a	nd.	310.65 ± 10.42 a	468.98 ± 34.65 b
	*Botrytis*	85.95 ± 3.03 b	nd.	49.40 ± 2.05 b	326.88 ± 11.25 b
	*Fusarium*	88.31 ± 2.42 b		45.81 ± 3.17 b	
	*Alternaria*	84.47 ± 3.84 b		54.65 ± 4.05 b	

Note: Values in the same row and subtable not sharing the same letters are significantly different at *p* < 0.05 in the two-sided test of equality for column means. Cells with no letters are not included in the test. Tests assume equal variances, HPLC was not carried out for *Alternaria*- and *Fusarium*-infected plants, nd.: not detected.

**Table 3:** Pearson correlation between the variables: total polyphenolic content (TPC) measured by two methods, antioxidant capacity (FRAP), anthocyanin content (TMA), infection percentages and the genotypes studied.

Pearson correlation coefficients in the case of *Alternaria*-infected plants							
	Phenophase	TPC	FRAP	Infection %	Genotype		
Phenophase	1	−0.104	−0.200 **	0.646 **	0		
TPC		1	0.830 **	−0.268	0.173		
FRAP			1	−0.175	0.140		
Infection %				1	−0.389 *		
Genotype					1		
Pearson correlation coefficients in the case of the *Fusarium*-infected plants							
	Phenophase	TPC	FRAP	Infection %	Genotype		
Phenophase	1	−0.095	−0.188	−0.531 **	0		
TPC		1	0.835 **	0.173	0.202 *		
FRAP			1	0.028	0.211 *		
Infection %				1	−0.129		
Genotype					1		
Pearson correlation coefficients in the case of the *Botrytis*-infected plants							
	Phenophase	TPC	FRAP	Infection %	Genotype	HPLC–TPC	HPLC–TMA
Phenophase	1	−0.096	−0.160	−0.974 **	0	−0.597 **	−0.462 **
TPC		1	0.834 **	0.022	0.208 *	0.453 **	0.462 **
FRAP			1	−0.246	0.202 *	0.472 **	0.458 **
Infection %				1	0.017	0.762 **	0.486 **
Genotype					1	0.526 **	0.519 **
HPLC–TPC						1	0.858 **
HPLC–TMA							1

* Correlation is significant at the *p* < 0.05 level (2-tailed). ** Correlation is significant at the *p* < 0.01 level (2-tailed).

**Table 4:** Putative identification of compounds of the purple NIL at two phenophases at Week 0 and after 13 dpi: control (mock-infected) and *Botrytis*-infected fruits.

	Purple			Red from Purple		
µg/g DW	Week 0	Control	*Botrytis*	Week 0	Control	*Botrytis*
Vitamin C	5526.84 ± 651.41 a	1792.47 ± 368.54 b	7023.42 ± 853.76 a	11,335.50 ± 1552.46 a	9819.50 ± 333.50 a	12,446.18 ± 496.94 a
Total Polyphenolics	2354.90 ± 247.23 a,b	2485.66 ± 217.84 a	1814.64 ± 59.41 b	1036.990 ± 330.23 a	732.03 ± 14.34 a	1017.70 ± 396.40 a
Total Anthocyanins	1027.08 ± 104.47 a	1005.64 ± 80.47 a	271.26 ± 46.96 b	105.82 ± 50.51 a	17.81 ± 4.33 a	nd.
delphinidin-3-rutinoside-glucoside	46.57 ± 1,27 a	60.47 ± 7.74 a	7.75 ± 1.50 b	8.85 ± 4.76	nd.	nd.
delphinidin-3-rutinoside	6.55 ± 1,27 a	6.05 ± 0.29 a	nd.	nd.	nd.	nd.
delphinidin-3-caffeoylrutinoside-5-glucoside	54.02 ± 8.18 a	88.69 ± 8.18 b	5.75 ± 2.77 c	8.20 ± 4.18	nd.	nd.
delphinidin-3-ciscoumaroylrutinoside-5-glucoside	45.11 ± 1.19 a	12.92 ± 0.85 b	6.18 ± 1.04 c	4.99 ± 2.54	nd.	nd.
delphinidin-3-transcoumaroylrutinoside-5-glucoside	840.00 ± 48.15 a	687.89 ± 72.69 a	248.63 ± 45.21 b	78.89 ± 37.34 a	17.81 ± 4.33 a	4.26 ± 0.16 a
delphinidin-3-feruoylrutinoside-5-hexose	29.06 ± 4.76 a	36.18 ± 2.66 a	nd.	2.47 ± 1.28	nd.	nd.
synergic acid diglucoside	30.78 ± 2.05 a	78.12 ± 7.32 b	55.64 ± 3.03 c	29.47 ± 1.79 a	12.70 ± 1.64 b	23.16 ± 2.06 a
coumaric acid hexose	16.21 ± 2.14 a	12.82 ± 1.22 a	5.04 ± 1.50 b	10.05 ± 0.30 a	10.39 ± 1.23 a	1.04 ± 0.07 b
caffeoyl dihexose	nd.	nd.	1.43 ± 0.37	1.95 ± 0.98 a,b	0.64 ± 0.08 a	4.68 ± 0.56 b
caffeoyl hexose	711.42 ± 48.02 a	431.06 ± 23.09 b	704.81 ± 102.03 a	75.05 ± 8.64 a	21.17 ± 4.45 b	108.60 ± 4.26 c
caffeoyl quinic acid derivative A	1.26 ± 0.07 a	42.72 ± 3.70 b	29.92 ± 2.70 c	23.44 ± 1.38	nd.	nd.
coumaroyl quinic acid	26.60 ± 1.98 a	21.75 ± 2.66 b	7.79 ± 1.34 c	5.70 ± 0.18 a	8.31 ± 0.36 b	4.75 ± 0.23 c
caffeoyl quinic acid derivative B	84.55 ± 6.00 a	12.23 ± 0.58 b	3.91 ± 1.28 c	21.85 ± 10.93 a	nd.	nd.
catechin	43.58 ± 2.42 a	207.95 ± 1.41 b	193.73 ± 25.44 b	69.89 ± 9.92 a	13.67 ± 0.85 b	125.46 ± 5.39 c
naringenin dihexose	121.54 ± 6.74 a	72.07 ± 2.45 b	35.21 ± 1.45 c	50.62 ± 3.97 a	36.14 ± 4.40 b	28.12 ± 2.78 b
chlorogenic acid	21.40 ± 3.00 a	56.37 ± 1.34 b	127.50 ± 5.96 c	124.68 ± 20.84 a	88.90 ± 13.50 a	17.51 ± 4.38 b
p-coumaric acid	nd.	nd.	39.93 ± 5.91	29.62 ± 3.14 a	16.07 ± 1.43 b	nd.
ellagic acid	6.70 ± 1.21 a	10.21 ± 0.44 b	nd.	nd.	nd.	nd.
cis-coumaric acid	34.13 ± 2.27 a	86.26 ± 8.81 b	21.92 ± 4.76 a	31.01 ± 0.74 a	87.22 ± 12.78 b	84.85 ± 1.35 b
trans-coumaric acid	26.38 ± 0.95 a	70.40 ± 2.81 b	18.34 ± 1.17 c	96.69 ± 6.55 a	93.46 ± 4.52 a,b	72.74 ± 1.14 b
dicoumaric acid hexose	73.23 ± 4.19 a	54.16 ± 3.47 a	25.73 ± 13.08 b	5.06 ± 0.86 a	nd.	1.31 ± 0.00 b
dicaffeoyl quninc acid	101.40 ± 7.31 a	240.44 ± 22.52 b	43.68 ± 4.47 a	97.08 ± 36.08 a	88.43 ± 12.19 a	185.17 ± 0.35 a
rutin	22.58 ± 0.62 a	15.91 ± 0.75 a	18.56 ± 4.79 a	11.90 ± 1.75 a	15.70 ± 1.29 a,b	20.61 ± 1.17 b
quercetin glucoside	51.55 ± 4.06 a	19.46 ± 1.83 b	33.14 ± 13.18 a,b	nd.	nd.	7.36 ± 0.37
dicaffeoyl glucoside	18.73 ± 0.51 a	12.34 ± 0.53 a	11.43 ± 7.20	7.82 ± 0.86 a	2.07 ± 0.26 b	9.59 ± 0.13 a
apigenin	nd.	nd.	2.85 ± 0.75	23.89 ± 1.10 a	20.42 ± 1.86 a	3.27 ± 0.54 b
luteolin diglucoside	13.42 ± 0.93 a	24.76 ± 1.58 b	5.22 ± 1.13 c	8.59 ± 1.41 a	18.51 ± 0.71 b	5.30 ± 1.86 a

Note: Values in the same row and subtable not sharing the same letters are significantly different at *p*< 0.05 in the two-sided test of equality for column means. Cells with no letters are not included in the test. Tests assume equal variances, nd.: not detected.

**Table 5:** Putative identification of compounds of the green NIL at two phenophases at Week 0 and after 13 dpi: Control (mock-infected) and *Botrytis*-infected fruits.

	Green			Red from Green		
µg/g DW	Week 0	Control	*Botrytis*	Week 0	Control	*Botrytis*
Vitamin C	3977.54 ± 547.09 a	2255.95 ± 433,82 a	9923.95 ± 939.88 b	10,941.23 ± 462.80 a	9587.57 ± 427.71 a	5487.04 ± 542.50 b
Total Polyphenolics	1601.22 ± 215.68 a	784.42 ± 27.86 b	911.28 ± 17.24 b	1264.37 ± 198.14 a	316.88 ± 9.68 b	326.88 ± 22.5 b
synergic acid diglucoside	25.00 ± 2.60 a	30.60 ± 3.63 a	21.19 ± 1.50 a	30.52 ± 1.69 a	10.28 ± 2.99 b	38.05 ± 3.93 a
coumaric acid hexose	76.02 ± 10.28 a	21.47 ± 2.84 b	22.94 ± 3.92 b	20.74 ± 1.23 a	8.61 ± 0.93 b	6.24 ± 2.14 b
caffeoyl dihexose	nd.	nd.	4.31 ± 0.32	4.31 ± 0.32 a	0.52 ± 0.07 b	4.23 ± 0.68 a
caffeoyl hexose	783.81 ± 67.44 a	297.54 ± 30.88 b	82.92 ± 12.60 c	154.77 ± 21.38 a	21.14 ± 2.43 b	97.68 ± 7.56 c
caffeoyl quinic derivative A	23.82 ± 4.52 a	19.54 ± 2.02 a	16.56 ± 0.43 a	10.64 ± 1.63 a	5.29 ± 1.31 b	nd.
Coumaroylquinic acid	30.02 ± 1.38 a	nd.	10.30 ± 2.56 b	11.39 ± 1.32 a	9.04 ± 1.55 a	3.30 ± 0.74 b
caffeoyl quinic acid derivative B	48.62 ± 0.70 a	46.40 ± 4.78 a	15.16 ± 4.67 b	22.48 ± 2.33	nd.	nd.
catechin	33.84 ± 2.79 a	140.04 ± 10.19 b	74.80 ± 7.81 b	143.51 ± 9.97 a	27.38 ± 3.8 a,b	170.09 ± 10.33 a
naringenin dihexose	64.26 ± 5.40 a	53.50 ± 3.81 a	91.21 ± 8.21 b	88.91 ± 10.51 a	18.42 ± 1.26 b	27.70 ± 3.00 b
chlorogenic acid	44.12 ± 1.79 a	49.93 ± 6.58 a	102.99 ± 18.5 b	150.08 ± 18.81 a	46.17 ± 1.71 b	10.75 ± 2.12 b
p-coumaric acid	nd.	nd.	45.17 ± 5.01	54.90 ± 2.83 a	5.36 ± 0.65 b	nd.
ellagic acid	12.25 ± 2.36 a	18.55 ± 3.41 a	8.70 ± 1.17 a	nd.	nd.	nd.
cis-coumaric acid	nd.	nd.	nd.	nd.	nd.	43.74 ± 13.80
trans-coumaric acid	nd.	nd.	nd.	nd.	nd.	37.31 ± 13.19
dicoumaric acid hexose	nd.	nd.	nd.	nd.	nd.	3.37 ± 2.02
dicaffeoyl quninc acid	47.15 ± 3.39 a	201.56 ± 7.66 b	nd.	nd.	126.88 ± 26.27 a	103.56 ± 22.40 a
rutin	nd.	nd.	nd.	nd.	nd.	22.05 ± 3.89
quercetin glucoside	68.66 ± 3.41 a	60.44 ± 2.93 a	11.84 ± 3.78 b	22.95 ± 2.56 a	nd.	10.66 ± 1.08 b
dicaffeoyl glucoside	nd.	nd.	1.98 ± 0.83	nd.	nd.	6.82 ± 1.70
apigenin	nd.	nd.	16.32 ± 3.67	15.83 ± 1.69 a	20.42 ± 2.14 a	4.91 ± 2.16 b
luteolin diglucoside	6.94 ± 0.18 a	13.68 ± 1.33 b	9.78 ± 1.95 a,b	8.87 ± 0.75 a	38.96 ± 1.31 b	2.72 ± 0.63 c

Note: Values in the same row and subtable not sharing the same letters are significantly different at *p* < 0.05 in the two-sided test of equality for column means. Cells with no letters are not included in the test. Tests assume equal variances; nd.: not detected. Total Anthocyanin and individual anthocyanin compounds are left out from the table as they were not detected in either phenophase.

## Data Availability

All of the data is contained within the article.

## Abbreviations

The following abbreviations are used in this manuscript:

*A. alternata*	*Alternaria alternata*
ANOVA	Analysis of Variance
*B. cinerea*	*Botrytis cinerea*
BR	Biological Ripeness
CGA	Chlorogenic acid
dpi	days post infection
ER	Economical Ripeness
*F. avenaceum*	*Fusarium avenaceum*
*F. culmorum*	*Fusarium culmorum*
*F. solani*	*Fusarium solani*
FRAP	Ferric Reducing Ability of Plasma
HPLC	High-Performance Liquid Chromatography
G	Green
Gt	Genotype
MANOVA	Multivariate Analysis of Variance
MG	Mature Green
NILs	Near Isogenic Lines
nd.	not detected
ns	not significant
P	Purple
*P. capsici*	*Phytophthora capsici*
PDA	Potato Dextrose Agar
Pp	Phenophase
qPCR	quantitative Polymerase Chain Reaction
RG	Red from Green
ROS	Reactive Oxygen Species
RP	Red from Purple
RR	Red Ripe
TFC	Total Flavonoid Content
TMA	Total Monomeric Anthocyanin Content
TPC	Total Polyphenolic Content
